# ASC-J9 Blocks Cell Proliferation and Extracellular Matrix Production of Keloid Fibroblasts through Inhibiting STAT3 Signaling

**DOI:** 10.3390/ijms23105549

**Published:** 2022-05-16

**Authors:** Yi-Kai Hong, Chen-Han Wu, Yu-Chen Lin, Yu-Lun Huang, Kuo-Shu Hung, Tsung-Pin Pai, Yen-Ting Liu, Tzu-Chi Chen, Hardy Chan, Chao-Kai Hsu

**Affiliations:** 1 Department of Dermatology, National Cheng Kung University Hospital, College of Medicine, National Cheng Kung University, Tainan 701, Taiwan; jack810325@gmail.com (Y.-K.H.); z31410@gmail.com (Y.-C.L.); i54091238@gs.ncku.edu.tw (Y.-L.H.); 2 International Center for Wound Repair and Regeneration (iWRR), National Cheng Kung University, Tainan 701, Taiwan; 3 Allianz Pharmascience, Ltd. (Now AnnJi Pharmaceutical, Co., Ltd.), Taipei 100, Taiwan; boudient@gmail.com (C.-H.W.); pai.pai@ajpharm.com (T.-P.P.); yen-ting.liu@ajpharm.com (Y.-T.L.); zuki.chen@ajpharm.com (T.-C.C.); hwchan@rocketmail.com (H.C.); 4 Department of Surgery, National Cheng Kung University Hospital, College of Medicine, National Cheng Kung University, Tainan 701, Taiwan; h3624917@gmail.com; 5 Institute of Clinical Medicine, College of Medicine, National Cheng Kung University, Tainan 701, Taiwan

**Keywords:** keloid fibroblast, ASC-J9, STAT3, cell proliferation, ECM production

## Abstract

Keloids are a fibrotic skin disorder caused by abnormal wound healing and featuring the activation and expansion of fibroblasts beyond the original wound margin. Signal transducer and activator of transcription 3 (STAT3) has been found to mediate the biological functions of keloid fibroblasts (KFs). Therefore, we aimed to demonstrate whether ASC-J9, an inhibitor of STAT3 phosphorylation, can suppress the activation of KFs. Western blotting results showed that ASC-J9 inhibited the levels of COL1A1 and FN1 proteins, which were upregulated in KFs, by decreasing the expression of pSTAT3 and STAT3. RNA sequencing and in vitro studies further demonstrated that ASC-J9 treatment of KFs reduced cell division, inflammation, and ROS generation, as well as extracellular matrix (ECM) synthesis. ELISA assays verified that ASC-J9 treatment significantly mitigated IL-6 protein secretion in KFs. Transmission electron microscopy images revealed that ASC-J9 induced the formation of multilamellar bodies in KFs, which is associated with autophagy-related signaling. These results suggested that inhibiting a vicious cycle of the ROS/STAT3/IL-6 axis by ASC-J9 may represent a potential therapeutic approach to suppress cell proliferation and ECM production in KFs.

## 1. Introduction

Keloids result from abnormal wound healing. By definition, keloids extend beyond the margins of the original wound and frequently cause discomfort from pain and itching [1]. The incidence of keloids in Africans, Asians, and Hispanics ranges from 4.5% to 16% and is greatly elevated for pregnant women, teenagers, and young adults [2]. The histopathological features of keloids include dense hyalinized keloidal collagen deposition, inflammation, increased cellularity, and α-smooth muscle actin (α-SMA) expression [3]. Keloid fibroblasts (KFs) that are positive for the myofibroblast marker α-SMA excessively produce extracellular matrix (ECM) and display greater activity of proliferation, migration, and invasion than normal fibroblasts (NFs) [4,5,6,7]. Even though multiple therapies, including surgical excision and radiation, have been established, keloids are still likely to recur after treatment [8]. Therefore, it is essential to develop novel treatments.

Signal transducer and activator of transcription 3 (STAT3) is a transcription factor in the STAT protein family. Stimulation by cytokines, inflammatory factors, growth factors, carcinogens, and stress activates JAK, SRC, c-ABL, and MAPKs (p38, ERK, and JNK), which can further phosphorylate Tyr-705 in the STAT3 transactivation domain [9]. Phosphorylated STAT3 dimerizes, and its translocation to the nucleus mediates the transcription of the target genes [10]. STAT3 is involved in several human diseases, including asthma, diabetes, neuron degeneration, depression, cancer, and tissue fibrosis; it also plays a role in fundamental biological functions such as proliferation, migration, differentiation, and ECM deposition [11,12]. STAT3 expression and phosphorylation are increased in keloids, whereas the knockdown of STAT3 in KFs reduces their capacities of collagen synthesis, proliferation, and migration [13,14]. This makes STAT3 a potential therapeutic target for keloids.

ASC-J9, a curcumin analog named dimethylcurcumin, suppresses proliferation and invasion in prostate cancer by inhibiting the phosphorylation of STAT3 (pSTAT3) [15]. Here, we propose that ASC-J9 can inhibit the activation of KFs by targeting STAT3 signaling. First, we demonstrated that ASC-J9 restrained the levels of COL1A1 and FN1 proteins, which were highly expressed in KFs, by decreasing the expression of pSTAT3 and STAT3. Transcriptomic analysis by RNA sequencing and in vitro studies demonstrated that ASC-J9 treatment significantly reduced cell proliferation, ECM synthesis, reactive oxygen species (ROS) generation, and IL-6 production in KFs. Transmission electron microscopy (TEM) identified the formation of multilamellar bodies after ASC-J9 administration, indicating that the KFs may undergo autophagy. These findings illustrated that ASC-J9 may be a potential drug against cell proliferation, ECM synthesis, inflammation, and ROS generation in KFs by interrupting STAT3 signal transduction.

## 2. Results

### 2.1. ASC-J9 Inhibits ECM Synthesis and Cell Proliferation in Keloid Fibroblasts

**Figure 1:**
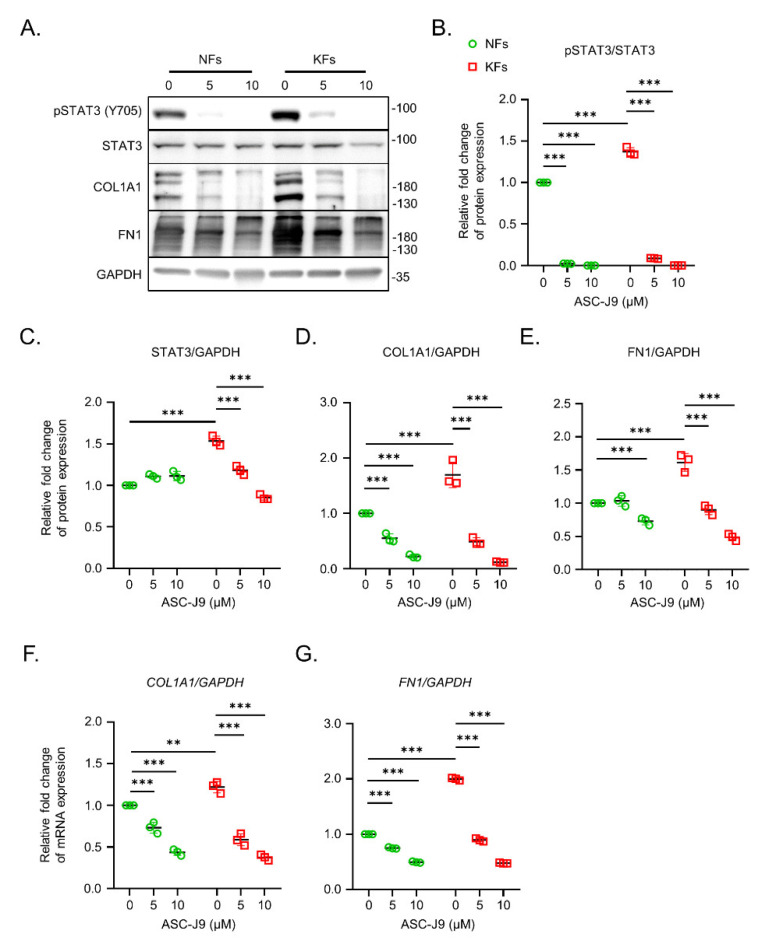
The blockage of ASC-J9 in extracellular matrix synthesis of normal fibroblasts and keloid fibroblasts. (**A**) The protein levels of pSTAT3 (Y705), STAT3, COL1A1, FN1, and GAPDH in NFs (*n* = 3) and KFs (*n* = 3) treated with ASC-J9 at various concentrations were examined by Western blotting. (**B**–**E**) The intensity of pSTAT3 expression relative to STAT3 was calculated on the basis of the results of Western blotting. The intensity of STAT3, COL1A1, and FN1 expression relative to GAPDH was also quantified. The relative fold change in protein expression in each group was normalized to that in NFs without ASC-J9 treatment. (**F**,**G**) The mRNA levels of *COL1A1* and *FN1* in NFs (*n* = 3) and KFs (*n* = 3) treated with 0, 5, or 10 μM ASC-J9 for 24 h, detected by qPCR. The relative fold change of mRNA expression in each group was normalized to that in NFs without ASC-J9 treatment. NFs, normal fibroblasts; KFs, keloid fibroblasts. ** *p* < 0.01, *** *p* < 0.001. *p*-values were determined by two-way analysis of variance.

First, the Western blotting results of keloids and the adjacent normal skin from the same subjects revealed that the expression levels of STAT3, COL1A1, and FN1 were increased in keloids relative to those in normal skin. Nevertheless, pSTAT3 expression was reduced in the keloids (Supplementary Figure S1A,B). KFs contribute to keloid progression through an increase in the excessive accumulation of extracellular matrix [16]. Previous studies found the expression of both pSTAT3 and STAT3 to be higher in KFs than in NFs, suggesting an important role of the STAT3 pathway in the biological functions of KFs [13,14]. Therefore, KFs and NFs were isolated from the keloid lesions and the adjacent non-lesional skins individually, and were subjected to the following studies. Importantly, the expression levels of pSTAT3 and STAT3 were increased in KFs relative to those in NFs. Treatment with ASC-J9 for 24 h significantly reduced the expression of pSTAT3 and STAT3 of KFs in a dose-dependent manner (Figure 1A–C). In addition, the expression levels of collagen Type I (COL1A1) and fibronectin (FN1) at the mRNA and protein levels were upregulated in KFs relative to those in NFs. Interestingly, treatment with 10 μM ASC-J9 significantly reduced the mRNA (Figure 1F,G) and protein expression (Figure 1A,D,E) of COL1A1 and FN1 in KFs.

**Figure 2:**
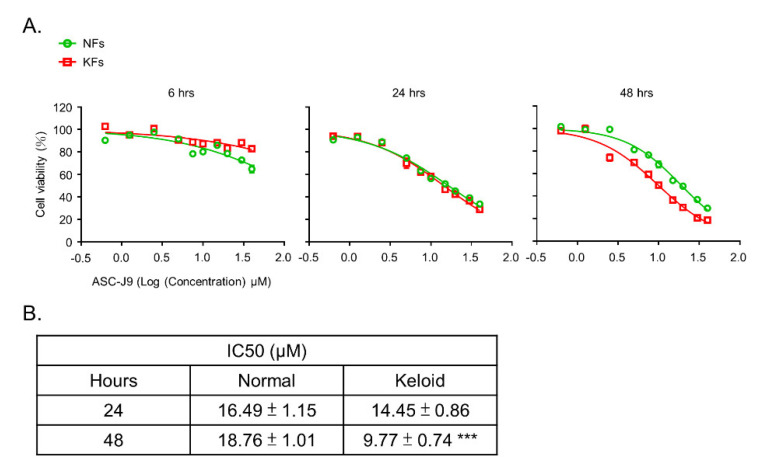
The inhibitory effect of ASC-J9 on the proliferation of normal fibroblasts and keloid fibroblasts. (**A**) The cell viability (%) of NFs (*n* = 3) and KFs (*n* = 3) treated with ASC-J9 for 6, 24, or 48 h was examined by MTT assays. (**B**) IC50 values at 24 or 48 h in NFs and KFs are shown. NFs, normal fibroblasts; KFs, keloid fibroblasts. *** *p* < 0.001. *p*-values were determined by unpaired two-tailed Student’s *t*-test.

To verify the inhibitory effect of ASC-J9 on cell viability, a functional evaluation with an MTT assay was performed. ASC-J9 treatment at a high dose blocked cell viability at 48 h (Figure 2A). At 24 h, there was no difference in IC50 between the NFs (16.49 ± 1.15 μM) and the KFs (14.45 ± 0.86 μM). At 48 h, however, IC50 was significantly lower in the KFs (9.77 ± 0.74 μM) than in the NFs (18.76 ± 1.01 μM). These results suggest that ASC-J9 treatment for 48 h can reduce the cell viability of NFs and KFs (Figure 2B).

### 2.2. RNA-Seq Reveals the Inhibitory Effect of ASC-J9 on Inflammation, ROS Generation, and Fibrosis in Keloid Fibroblasts

**Figure 3:**
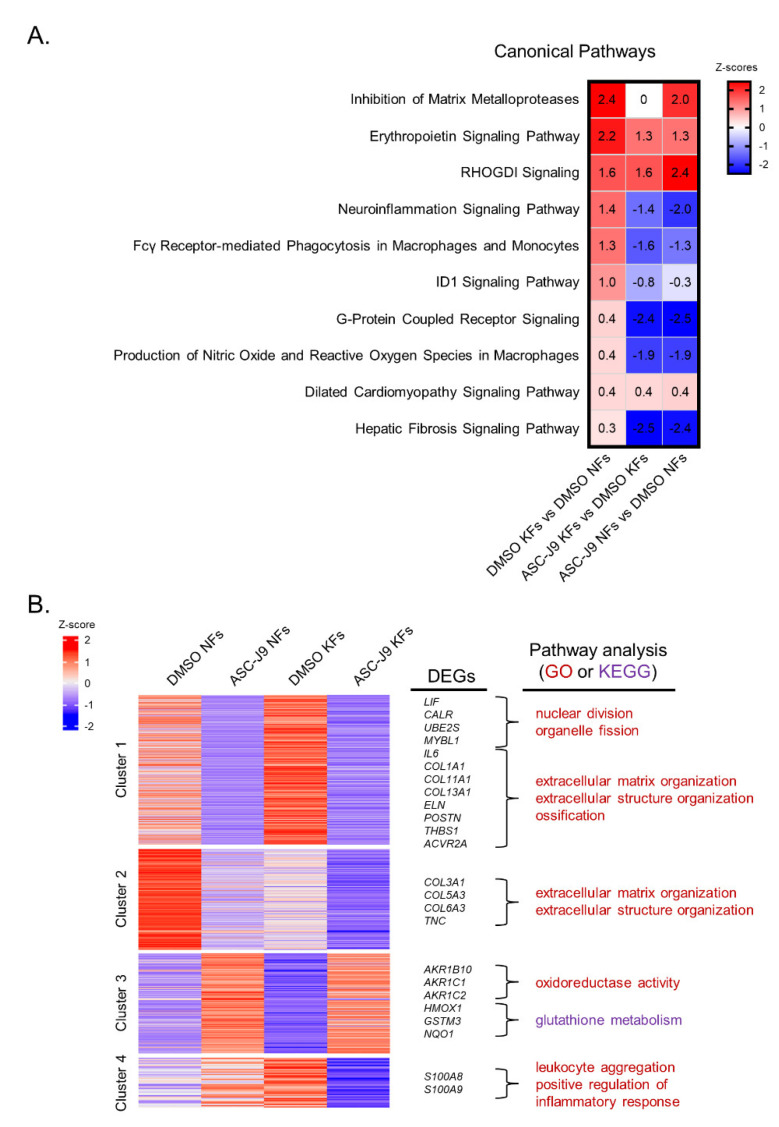
The transcriptomic profiling of normal and keloid fibroblasts, showing the inhibitory effect of ASC-J9 on inflammation, oxidative stress, and fibrosis. (**A**) Transcriptomic profiling of NFs (*n* = 1) and KFs (*n* = 1) treated with DMSO or 10 μM ASC-J9 for 24 h was performed by RNA sequencing. Canonical pathways were analyzed by Ingenuity Pathway Analysis (IPA) following identification of DEGs. (**B**) Cluster methods were carried out by the tidyverse package to explore gene expression patterns. The gene ontology biological processes of DEGs in each cluster were analyzed by the clusterProfiler package. DEGs, differentially expressed genes; NFs, normal fibroblasts; KFs, keloid fibroblasts.

To understand the global transcriptome effect of ASC-J9 on KFs, mRNA extracted from NFs or KFs treated with DMSO or ASC-J9 was subjected to RNA-seq. Analysis of the canonical pathways by Ingenuity Pathway Analysis (IPA) showed that the increased fibrotic enrichment pathways, including the inhibition of matrix metalloproteases pathway, the ID1 signaling pathway, and the hepatic fibrosis signaling pathway, were found in DMSO KFs compared with DMSO NFs [17,18,19]. Inflammatory and ROS pathways containing the neuroinflammation signaling pathway, Fcγ receptor-mediated phagocytosis in macrophages and monocytes, and production of nitric oxide and ROS in macrophages were also enriched in DMSO KFs versus DMSO NFs. The G-protein coupled receptor signaling and dilated cardiomyopathy signaling pathways were also enhanced in DMSO KFs that were associated with cardiac fibrosis [20,21]. ASC-J9 treatment of NFs and KFs blocked the neuroinflammation signaling pathway, Fcγ receptor-mediated phagocytosis, G-protein coupled receptor signaling, the production of nitric oxide and ROS, and the hepatic fibrosis signaling pathway (Figure 3A). To identify potential differences in transcriptional responses to ASC-J9 treatment for NFs and KFs, cluster methods were used to explore the gene expression patterns with statistically significant differences [22]. Interaction analyses of NF and KF datasets revealed four clusters of genes that were differentially regulated by ASC-J9 treatment. Cluster 1 contained a set of genes that included *LIF*, *CALR*, *UBE2S*, *MYBL1*, *IL6*, *COL1A1*, *COL11A1*, *COL13A1*, *ELN*, *POSTN*, *THBS1*, and *ACVR2A*, which were related to cell division and ossification, and were upregulated in the DMSO KFs relative to the DMSO NFs, but ASC-J9 in both cells exhibited decreased expression. Cluster 2 represented regenerative gene sets such as *COL3A1*, *COL5A3*, *COL6A3*, and *TNC* that were downregulated in DMSO KFs relative to DMSO NFs and were further reduced in ASC-J9-treated NFs and KFs [23,24,25,26]. In Cluster 3, gene sets including *AKR1B10*, *AKR1C1*, *AKR1C2*, *HMOX1*, *GSTM3*, and *NQO1*, which are associated with oxidoreductase activity and glutathione metabolism, and regulate antioxidant defense, were increased in ASC-J9-treated NFs and KFs relative to the DMSO groups [27]. Cluster 4 displayed pro-inflammatory gene sets, such as *S100A8* and *S100A9*, which were increased in the DMSO KFs relative to the DMSO NFs but were diminished in ASC-J9 KFs relative to the DMSO KFs (Figure 3B). To summarize, ASC-J9 inhibits biological pathways including cell proliferation, fibrosis, and inflammation in KFs.

### 2.3. ASC-J9 Treatment Regulates Oxidative Stress Homeostasis and Inflammatory Responses

**Figure 4:**
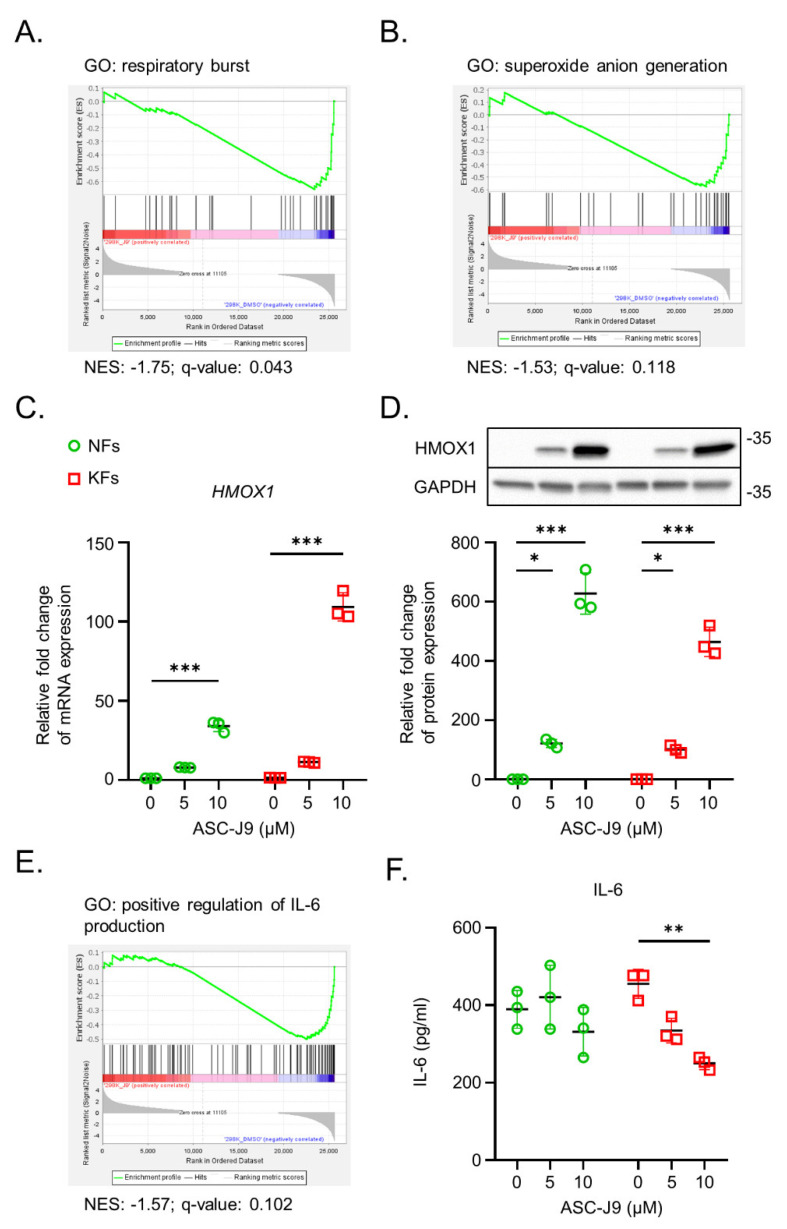
The regulation of ASC-J9 of the oxidative and inflammatory responses of normal fibroblasts and keloid fibroblasts. (**A**,**B**) The biological pathways related to ROS were analyzed by GSEA. (**C**,**D**) The mRNA and protein levels of HMOX1 expression in NFs (*n* = 3) and KFs (*n* = 3) treated with 0, 5, or 10 μM ASC-J9 for 24 h were detected by qPCR and Western blotting. The relative fold change in mRNA and protein expression in each group was normalized to that in NFs without ASC-J9 treatment. (**E**) The biological processes related to inflammation were indicated by GSEA. (**F**) IL-6 protein of NFs (*n* = 3) and KFs (*n* = 3) in a conditioned medium after treatment with 0, 5, or 10 μM of ASC-J9 for 24 h was detected by ELISA. The relative fold change in protein expression of each group was normalized to that in NFs without ASC-J9 treatment. GSEA, gene set enrichment score; ROS, reactive oxygen species; ELISA, enzyme-linked immunosorbent assay; NFs, normal fibroblasts; KFs, keloid fibroblasts. * *p* < 0.05, ** *p* < 0.01, *** *p* < 0.001. *p*-values were determined by two-way analysis of variance.

Curcumin inhibits intracellular ROS generation, which can suppress tumor cell growth [28,29,30]. ROS-activated STAT3 drives the expression of *IL6* gene by binding to its promotor [31]. It is speculated that ASC-J9, a curcumin analog that can inhibit STAT3, can inhibit the production of ROS by keloid fibroblasts. GSEA analysis showed that ASC-J9 treatment decreased respiratory burst and superoxide anion generation in keloid fibroblasts (Figure 4A,B). ASC-J9 treatment also enhanced the mRNA and protein levels of HMOX1 expression in NFs and KFs, which has been shown to protect cells against ROS formation (Figure 4C,D) [32]. ROS triggers rapid inflammatory responses [33]. Hence, the inhibitory ability of ASC-J9 in inflammation was further explored in KFs by ELISA. ASC-J9 treatment of KFs showed a reduction in the positive regulation of IL-6 (Figure 4E). Further confirmation with the ELISA assay showed that ASC-J9 treatment at 10 μM significantly reduced IL-6 protein secretion in KFs (Figure 4F). In brief, ASC-J9 treatment enhances antioxidant responses and reduces IL-6 production in KFs.

### 2.4. ASC-J9 Induces the Formation of Multilamellar Bodies in Keloid Fibroblasts

**Figure 5:**
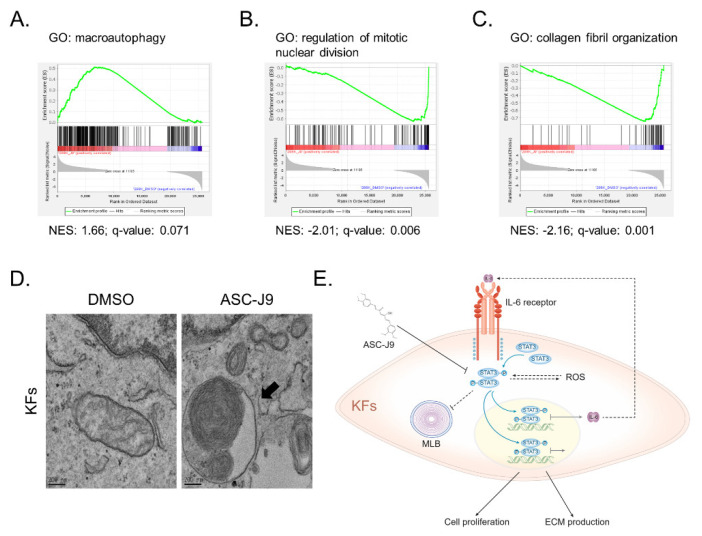
The formation of multilamellar bodies induced by ASC-J9 in keloid fibroblasts. (**A**–**C**) The biological pathways related to autophagy, cell proliferation, and ECM synthesis were identified by GSEA. (**D**) The structure of multilamellar bodies in KFs (*n* = 3) treated with 10 μM ASC-J9 for 24 h was examined by TEM. (**E**) A proposed mechanism by which ASC-J9 affects the biological functions of KFs is shown. ECM, extracellular matrix; GSEA, gene set enrichment score; TEM, transmission electric microscopy; NFs, normal fibroblasts; KFs, keloid fibroblasts.

To explore the mechanisms by which ASC-J9 inhibits cell proliferation and ECM synthesis, GSEA was used to identify the key pathways after ASC-J9 treatment of KFs. The results showed that ASC-J9 treatment induced gene expression that was positively related to macroautophagy (Figure 5A) but negatively related to cell proliferation and ECM synthesis (Figure 5B,C). To evaluate the effect of ASC-J9 on the autophagy structure, transmission electron microscopy was applied. Interestingly, the formation of multilamellar bodies (MLBs) was found in KFs after ASC-J9 treatment (Figure 5D). MLB biogenesis is reportedly regulated by autophagy [34]. Autophagy is known to induce cell cycle arrest and ECM degradation [35,36,37]. Accordingly, ASC-J9 application reduces cell proliferation and ECM synthesis, and promotes MLB formation in KFs.

## 3. Discussion

KFs play a major role in keloid formation and have a higher proliferative rate and synthesis of ECM than NFs [38,39]. Our results verified that the mRNA and protein levels of COL1A1 and FN1 were increased in KFs. The expression of pSTAT3 and STAT3 was also augmented in KFs relative to that in NFs. pSTAT3 can form a dimer through reciprocal Src homology 2 (SH2)–phosphotyrosine interactions and can translocate to the nucleus, driving pro-fibrotic gene expression [12]. Previous studies found that pSTAT3 and STAT3 are overexpressed in keloid fibroblasts and that STAT3 interference by siRNA, Cucurbitacin I, and AG490 inhibits cell proliferation, migration, and ECM production in KFs [13,40]. ASC-J9 is known for suppressing the invasion of prostate cancer cells by inhibiting STAT3 phosphorylation [15]. In this study, ASC-J9 treatment in KFs significantly diminished the pSTAT3 and STAT3 protein levels. Importantly, ASC-J9 treatment can also significantly inhibit cell growth, ECM production, inflammation, and ROS generation in KFs, according to the in vitro results and transcriptomic analysis. These findings demonstrate that STAT3 in KFs can be a crucial target in keloid treatment.

Other studies showed that pSTAT3 expression is enhanced in keloid tissues relative to that in normal skin from healthy subjects [13]. However, our Western blotting results showed that pSTAT3 expression was decreased in keloids relative to that in the adjacent normal skin from the same patients with keloids. It is possible that the adjacent normal skin appears to be a pro-fibrotic microenvironment with lymphocyte infiltration [41,42]. Under long-term IL-6 stimulation, unphosphorylated STAT3 (uSTAT3) was found to bind to NFκB to activate gene transcription through a mechanism completely distinct from that of pSTAT3 [43]. These complexes of uSTAT3 are different from the classical tyrosine phosphorylation that facilitates STAT3 dimerization to bind to the interferon-gamma activated sequence [44]. Our data showed that total STAT3 was found to be increased in both keloid tissues and fibroblasts. Both pSTAT3 and uSTAT3 possibly contribute to the aberrant biological functions seen in keloids, but by different mechanisms. Importantly, previous studies and our group demonstrated that pSTAT3 expression is increased in keloid fibroblasts [13]. These results indicated that the dysregulation of pSTAT3 in keloid fibroblasts could play a role in keloid development.

In response to oxidative injury and an inflammatory reaction, ROS was found to be significantly upregulated in KFs [45], similar to our transcriptomic results. ROS generation can further mediate cell proliferation in KFs [46] and the activation of fibroblasts [47]. Previous studies revealed that HMOX1 induction by curcumin has an antioxidant effect on human fibroblasts via redox signaling [48,49]. Heme oxygenase 1 (HMOX1) is known to be one of the most sensitive and dependable indicators of oxidative stress in cells. HMOX1 has a protective function against oxidative tissue injury [50]. In this study, ASC-J9, a curcumin derivative, also suppressed ROS signaling in KFs, probably by increasing the expression of the antioxidant *HMOX1* gene. Additionally, ASC-J9 administration also induced HMOX1 protein expression in NFs to a level that was even higher than that in KFs. Upregulation of HMOX1 can protect normal cells from oxidative damage and cellular senescence during stress [51,52]. Therefore, HMOX1 induction by ASC-J9 could play a protective role in decreasing oxidative stress and activation of NFs and KFs.

Translocation of STAT3 to the mitochondria was found to control the activation of the electron transport chain, a major source of ROS synthesis [53]. Inversely, a lack of mitochondrial STAT3 significantly reduces oxidative responses [54]. ROS-induced STAT3 activation promotes IL-6 expression, while the binding of IL-6 to its receptors also drives STAT3 signal transduction, which forms a vicious cycle [31]. IL-6 expression was found to be upregulated in KFs, which agrees with our data [55]. A previous study showed that IL-6 signaling plays an integral role in keloid pathogenesis [56]. Hence, our findings showed that targeting STAT3 with ASC-J9 repressed IL-6 expression, ECM synthesis, and cell proliferation in KFs. Collectively, ASC-J9 treatment blocks the biological functions of KFs, possibly by diminishing the ROS/STAT3/IL-6 axis (Figure 5E).

The IL-6/STAT3 signaling pathway plays a repressive role in autophagy [57]. GSEA showed that ASC-J9 treatment induced autophagy and reduced cell proliferation and ECM production in KFs. We further used electron microscopy to observe autophagy formation, which revealed that ASC-J9 treatment stimulated autophagy-regulated MLB formation in KFs [34]. Autophagy (self-eating) is a physiological degradative process that removes unnecessary or dysfunctional components to facilitate bioenergetic homeostasis through the fusion of autophagosomes and lysosomes in all eukaryotic cells, and it is triggered in response to various environmental stresses, and assists cellular survival [58]. The excessive degradation of cytoplasmic components with persistent autophagy contributes to cell death [59]. On the other hand, autophagy can promote the degradation of internalized collagen Type I [35,36]. Therefore, our studies revealed that ASC-J9 administration inhibited cell division and ECM production, and stimulated MLB formation in KFs.

We found that ASC-J9 treatment can also reduce the protein expression of COL1A1 and FN1 in NFs. However, the inhibitory effect of ASC-J9 on ECM proteins in NFs was less than that in KFs. In addition, the IC50 of NFs after ASC-J9 treatment for 48 h was twofold higher than that of KFs, suggesting less inhibition of ASC-J9 in the cell viability of NFs. These results revealed that KFs were more sensitive to ASC-J9 than NFs in terms of ECM production and cell viability. We could not exclude the possibility that ASC-J9 could impact physically biological functions in NFs to further interrupt the wound healing process or the skin structure. In the future, in vivo studies will be essential to carefully prove the suitable occasions for ASC-J9 application to the wounds of keloid patients. Alternatively, intradermal injection of ASC-J9 in formed keloids may be a better strategy to reduce the adverse effects on skin.

## 4. Materials and Methods

### 4.1. Keloid Patients

The keloids were diagnosed by dermatologists based on the clinicopathological features. Bulging, erythematous nodules were defined as keloid lesions, whereas regions that were at least 1.5 cm from active regions and that showed neither erythematous changse nor raised skin were defined as non-lesional keloid areas. The study was approved by the Institutional Review Board of the National Cheng Kung University Hospital, based on the Declaration of Helsinki (NCKUH B-BR-109-074). Written consent was obtained from all patients before surgery. Human skin samples obtained during surgery were immediately transported to the laboratory for fibroblast culturing and further experiments. The attributes of the patients from which tissue was sampled are shown in Table S1.

### 4.2. Primary Culture of Fibroblasts

Dermal fibroblasts were isolated from dermal tissue according to a previous publication [7,60]. Briefly, the epidermis and subcutaneous tissue were excised and removed from the sample tissue. The remaining dermis was sliced into roughly 1 mm^3^ fragments that were placed as explants in 10 cm culture dishes and supplemented with Dulbecco’s modified Eagle medium (Sigma-Aldrich, Burlington, MA, USA) containing 10% fetal bovine serum (Invitrogen, Waltham, MA, USA), 2 mM L-glutamine (Invitrogen, Waltham, MA, USA), 100 U/mL penicillin (Sigma-Aldrich, Burlington, MA, USA), and 100 μg/mL streptomycin (Sigma-Aldrich, Burlington, MA, USA). The cells were kept at 37 °C in a humidified incubator with 5% CO_2_. The growth medium was changed every 3 or 4 days. When fibroblast outgrowth became well established (as Passage 0), the explants were removed prior to subculturing of the monolayer. The fibroblasts were generally subcultured at a 1:3 ratio when the cells reached around 80% confluence. For the experiment, fibroblasts between the third and eighth passages were used.

### 4.3. Quantitative PCR (qPCR)

RNA was extracted by the RNeasy Mini Kit (QIAGEN, Hilden, Germany), then 2 μg of total RNA was reverse-transcribed to the complementary DNA using 200 ng of the oligo (dT) _15_ primer, 1 mM deoxynucleotides, 5X reverse transcriptase buffer, 20 units of RNase inhibitor, and 200 units of M-MLV reverse transcriptase (Thermo Fisher Scientific, Waltham, MA, USA). Next, 10 ng of complementary DNA was amplified and detected by the real-time PCR machine (StepOne Real-Time PCR System, Thermo Fisher Scientific, Waltham, MA, USA) with 400 nM of primers synthesized by Genomics (Genomics, New Taipei City, Taiwan) and SYBR Green Master Mix (Thermo Fisher Scientific, Waltham, MA, USA). The relative levels of mRNA expression were measured using the 2-ΔCT method, where ΔCT is equal to the CT of the target gene substracting the CT of the reference gene. Glyceraldehyde 3-phosphate dehydrogenase (GAPDH) was used as the reference gene. The primer sequences are listed in Table S2.

### 4.4. Western Blotting

Cell lysates were extracted by a modified RIPA buffer (150 mM NaCl, 1 mM EGTA, 50 mM Tris (pH 7.4), 10% glycerol, 1% Triton X-100, 1% sodium deoxycholate, 0.1% SDS, and a protease inhibitor cocktail) (Sigma-Aldrich, Burlington, MA, USA). After sonication, total protein (20 μg) was segregated by 10% sodium dodecyl sulfate-polyacrylamide gel electrophoresis (Protech, Taipei, Taiwan) and transferred to a polyvinylidene difluoride membrane (Merck, Darmstadt, Germany). The membrane was blocked using 5% non-fat dry milk (Anchor, Auckland, New Zealand) for 1 h at room temperature, hybridized with primary antibody overnight at 4 °C, and washed by PBST, before being incubated with horseradish peroxidase-conjugated secondary antibody (Invitrogen, Waltham, MA, USA) for 2 h at room temperature. An enhanced chemiluminescence (ECL) system (GE Healthcare Life Sciences, Buckinghamshire, UK) was used to detect the target proteins on Western blots. Band intensity was quantified using ImageJ software. The concentrations and product codes of the primary and secondary antibodies used for Western blotting in this study are listed in Table S3.

### 4.5. MTT Assay

Fibroblasts were seeded at a density of 5000 cells per well in 96-well plates with a culture medium. After ASC-J9 treatment at 0, 0.625, 1.25, 2.5, 5, 7.5, 10, 15, 20, 30, or 40 μM for 6, 24, or 48 h, 100 μL of 0.5 mg/mL MTT (Sigma-Aldrich, Burlington, MA, USA) was added to each well. After 3 h, the MTT solution was removed and 50 μL of DMSO (Sigma-Aldrich, Burlington, MA, USA) was added to each well, and the absorbance at 570 nm was detected by a Synergy HT reader (BioTek, Winooski, VT, USA) 5 min later.

### 4.6. RNA Library Preparation and Sequencing

The purified RNA from NFs (*n* = 1) and KFs (*n* = 1) treated with DMSO or ASC-J9 was used for the preparation of the sequencing library by the TruSeq Stranded mRNA Library Prep Kit (Illumina, San Diego, CA, USA) according to the manufacturer’s recommendations. Briefly, mRNA was purified from total RNA (1 µg) by oligo(dT)-coupled magnetic beads and fragmented into small pieces under an elevated temperature. The first-strand cDNA was synthesized using reverse transcriptase and random primers. After the generation of double-strand cDNA and adenylation on the 3′ ends of the DNA fragments, the adaptors were ligated and purified with the AMPure XP system (Beckman Coulter, Brea, CA, USA). The quality of the libraries was assessed by the Agilent Bioanalyzer 2100 system (Agilent Technologies, Santa Clara, CA, USA) and a real-time PCR machine (StepOne Real-Time PCR System, Thermo Fisher Scientific, Waltham, MA, USA). The qualified libraries were then sequenced on a NovaSeq 6000 platform (Illumina, San Diego, CA, USA) with 150 bp paired-end reads generated by Genomics BioSci & Tech, Co., Ltd.

### 4.7. Bioinformatic Analysis

The bases with low quality and sequences from adapters in the raw data were removed using the program Trimmomatic (version 0.39) [61]. The filtered reads were aligned to the reference genomes using Bowtie2 (version 2.3.4.1) [62]. A user-friendly software application, RSEM (version 1.2.28), was applied for the quantification of transcript abundance [63]. Differentially expressed genes (DEGs) were identified by EBSeq (version 1.16.0) [64]. As functional enrichment analysis of the Gene Ontology (GO) terms among the gene clusters was implemented with the clusterProfiler package (version 3.6.0) [65,66]. Canonical pathways among the gene clusters were analyzed by Ingenuity Pathway Analysis (QIAGEN) [67]. Gene set enrichment analysis was performed by GSEA software (version 4.1.0) [68]. Cluster methods were performed by the tidyverse package (version 1.3.0) to explore the gene expression patterns [22].

### 4.8. Enzyme-Linked Immunosorbent Assay (ELISA)

After ASC-J9 treatment at 0, 5, or 10 μM for 24 h, the conditioned medium of the fibroblasts was harvested. IL-6 expression was assayed by Human IL6 ELISA Kit (Arigo Biolaboratories, Hsinchu City, Taiwan). In brief, a series of diluent standards and 10X diluent samples were added into each well and then incubated for 1.5 h at 37 °C. After washing each well with a washing buffer for a total of five times, an antibody-conjugated buffer was added and then incubated for 1 h at 37 °C. After washing, an HRP-streptavidin solution was added and then incubated for 30 min at 37 °C in the dark. After washing, a TMB solution was added and then incubated for 15 min at 37 °C in the dark. A stopping solution was added to terminate the reaction. The absorbance at 450 nm was detected by a Synergy HT reader (BioTek, Winooski, VT, USA).

### 4.9. Transmission Electron Microscopy

The tissue was fixed in a fixative containing 2.5% glutaraldehyde and 3 mM CaCl_3_ in a 0.1 M cacodylate buffer for 1 h at room temperature. After a washing with a 0.1 M cacodylate buffer containing 3 mM CaCl_3_, the tissue was post-fixed for 40 min at 4 °C in a 0.1 M cacodylate buffer containing 1% osmium tetroxide and 1.5% potassium ferricyanide. Samples were washed briefly in distilled water, followed by gradual dehydration in a graded ethanol series of 70%, 90%, 95% (15 min per stage), and then 100% (three times and 30 min each time). The dehydrated samples were then infiltrated in stages with spur resin–ethanol solutions containing 50%, then 75%, and then 100% resin (1 h per stage). The infiltrated samples were left in 100% spur resin overnight. Next, the samples were embedded in the models with fresh resin and were polymerized at 70 °C for 24 h. The embedded cells were cut using an ultramicrotome (Leica Biosystems, Wetzlar, Germany) into 70-nm ultrathin sections by a diamond knife and were harvested on nickel grids. The grids were then post-stained with uranyl acetate and lead citrate. Transmission electron microscopy was carried out on a JEM-1400 (JEOL, Tokyo, Japan) at 120 keV equipped with a CCD camera system (Ultrascan, Los Angeles, CA, USA).

### 4.10. Statistical Analysis

Data were displayed as means ± standard errors of the mean (SEM) of independent experiments. Two-way analysis of variance and Student’s *t*-test were applied to calculate the statistical significance in GraphPad Prism 8.0 (GraphPad Software, San Diego, CA, USA). *p*-values of <0.05 were considered significant.

## 5. Conclusions

This study showed that ASC-J9 suppressed STAT3 signaling transduction, thereby resulting in the decreased expression of COL1A1 and FN1 proteins, which were upregulated on KFs in comparison with NFs. Furthermore, ASC-J9 inhibited the cell viability of KFs, which were more sensitive to ASC-J9 treatment than that of NFs. To demonstrate the underlying molecular mechanism, transcriptomic analysis was used to verify that ASC-J9 greatly mitigated biological pathways including cell proliferation, fibrosis, inflammation, and ROS generation in KFs. Functional studies further confirmed that ASC-J9 promoted HMOX1 expression, which is an antioxidant factor, and autophagy-regulated MLB formation, which negatively regulates cell proliferation and ECM synthesis. In addition, ASC-J9 administration abolished inflammatory IL-6 secretion in KFs. Taking these facts all together, we propose that the interaction of the ROS/STAT3/IL-6 axis provides deleterious feedback that results in the progression of KFs. ASC-J9 intervention can block this vicious cycle and can further obstruct ECM production and cell proliferation in KFs, followed by MLB formation (Figure 5E). Accordingly, ASC-J9 may have potential as a drug therapy against keloid development.

## Data Availability

RNA-seq data have been uploaded to the Gene Expression Omnibus (GEO) database under the accession code “GSE198095”. The data presented in this study are available on request from the corresponding author.

## Supplementary Materials

The following are available online at https://www.mdpi.com/article/10.3390/ijms23105549/s1.
