# Supplementary material for: ASC-J9 Blocks Cell Proliferation and Extracellular Matrix Production of Keloid Fibroblasts through Inhibiting STAT3 Signaling
Source: Int J Mol Sci. 2022 May 16;23(10):5549. doi: 10.3390/ijms23105549 (PMC9141592; doi:10.3390/ijms23105549)
Supplement: Supplementary file 1 [file ijms-23-05549-s001.zip › ijms-1715510-supplementary.pdf]

ASC-J9 blocks cell proliferation and extracellular matrix production of keloid fibroblasts through inhibiting STAT3 signaling

Yi-Kai Hong, Chen-Han Wu, Yu-Chen Lin, Yu-Lun Huang, Kuo-Shu Hung, Tsung-Pin Pai, Yen-Ting Liu, Tzu-Chi Chen, Hardy Chan, Chao-Kai Hsu

Supplementary materials

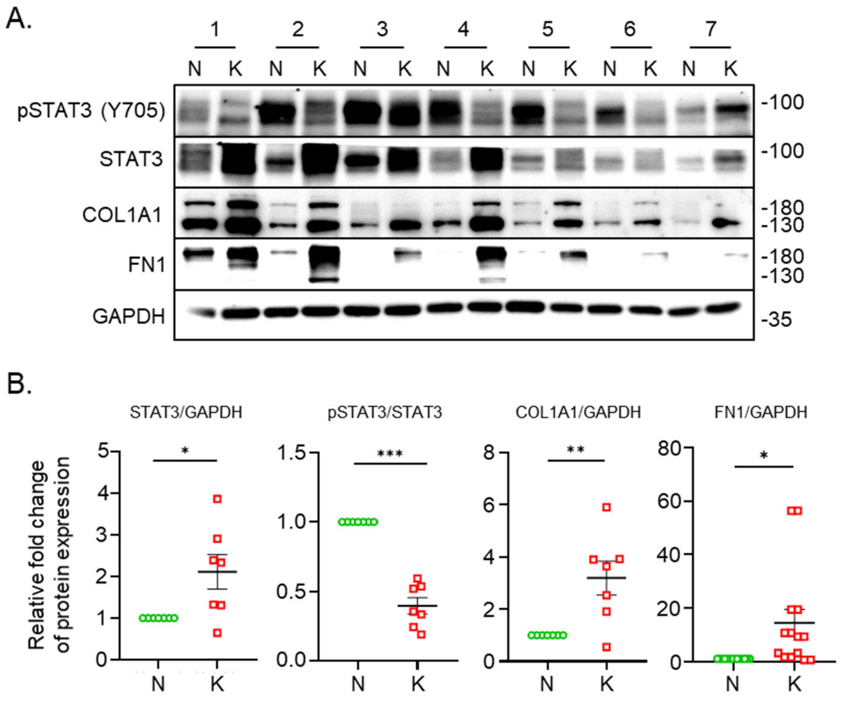

Supplementary Figure S1. The protein expression of STAT3, pSTAT3, COL1A1, and FN1 in normal and keloid tissues. (**A**) The protein levels of STAT3, pSTAT3, COL1A1, FN1, and GAPDH in each tissue are examined by Western blotting. (**B**) The intensity of STAT3, COL1A1, and FN1 expression relative to GAPDH is calculated based on the results of Western blotting. The intensity of p-STAT3 expression relative to STAT3 is

calculated. The ratio of each protein in keloid tissue relative to corresponding normal tissue is shown. N, normal tissue; K, keloid tissue. * $P < 0.05$, ** $P < 0.01$, *** $P < 0.001$. P-values are determined by unpaired two-tailed Student's t-test.

Supplementary Table S1. Characteristics of the individuals in this study

No.	Age (years)	Sex	Biopsy site	Duration (years)
1	57	Male	Abdomen	40
2	50	Male	Chest	10
3	73	Male	Chest	20
4	29	Male	Shoulder	15
5	41	Female	Chest	5
6	44	Female	Chest	7
7	35	Male	Shoulder	20
8	57	Female	Chest	20
9	35	Female	Arm	10
10	41	Female	Chest	5

Supplementary Table S2. List of sequences of primers used in real-time PCR

Gene	Species	Forward primer	Reverse primer
FN1	Human	TAGATGTACAGGCTGACAGAGAAG	AGAGACATGCTTGTTCCTCTGG
COL1A1	Human	CAAGGTGTTGTGCGATGACG	TTTCTTGGTCGGTGGGTGAC
HMOX1	Human	GTGGCGACAGTTGCTGTAGG	CGGTAAGGAAGCCAGCCAAG

Supplementary Table S3. The product catalog numbers and concentrations of primary antibodies used in Western blotting (WB)

Primary antibody, Company (Product catalog number)	Primary Ab raised species, isotype, concentration	MW (kD)	Secondary Ab concentration
			WB
Fibronectin (BD Biosciences, 610078)	Mouse IgG (WB 1:1000)	240	(1:5000)
COL1A1 (Novus Biologicals, NB600-408)	Rabbit IgG (WB 1:1000)	139	(1:5000)
Phospho-STAT3 (Tyr705) (Cell Signaling Technology, #9145)	Rabbit IgG (WB 1:2000)	79.86	(1:5000)
STAT3 (abcam, ab119352)	Mouse IgG (WB 1:1000)	79.86	(1:5000)
GAPDH (GeneTex, GTX100118)	Rabbit IgG (WB 1:10000)	36	(1:5000)
HMOX1 (abcam, ab13248)	Mouse IgG (WB 1:2000)	32	(1:5000)
